# Entangled Autopoiesis: Reframing Psychotherapy and Neuroscience Through Cognitive Science and Systems Engineering

**DOI:** 10.3390/brainsci15101032

**Published:** 2025-09-24

**Authors:** Dana Rad, Monica Maier, Zorica Triff, Radiana Marcu

**Affiliations:** 1 Centre of Research Development and Innovation in Psychology, Faculty of Educational Sciences, Aurel Vlaicu University of Arad, 310032 Arad, Romania; dana@xhouse.ro; 2 Department of Teacher Training, Technical University of Cluj-Napoca, 430122 Cluj-Napoca, Romania; zorica.triff@dspp.utcluj.ro

**Keywords:** autopoiesis, psychotherapy, clinical neuroscience, cognitive science, systems engineering, complex adaptive systems, artificial intelligence, therapeutic alliance

## Abstract

The increasing intersection of psychotherapy, cognitive science, neuroscience, and systems engineering beckons us to rethink what it means to talk the language of the human mind in the clinical setting. This position paper proposes the idea of entangled autopoiesis, a metatheoretical paradigm that addresses the mind and therapy not as linear processes but as self-organizing, adaptive processes enfolded across neural, cognitive, relational, and cultural domains. Psychotherapy, from this viewpoint, is less a corrective technique and more a zone of systemic integration, wherein resilience and meaning are co-created in the interaction of embodied brains, lived stories, and relational fields. Neuroscience informs us about plasticity and regulation; cognitive science emphasizes the embodied and extended nature of cognition; and systems engineering sheds light on feedback, emergence, and adaptive dynamics. Artificial intelligence appears as a double presence: as a metaphor for complexity and as a practical tool able to chart patterns below human sensibility. By adopting a complexity-aware epistemology, we advocate a relocation in clinical thinking—one recognizing the psyche as an autopoietic network, entangled with culture and technology and able to renew itself in therapeutic encounters. The implications for clinical methodology, therapist training, and future interdisciplinary research are discussed.

## 1. Introduction

The historical development of psychotherapy and neuroscience stands as a prime example of an interdisciplinary effort directed at understanding the most complicated aspects of the human mind. Ranging from psychoanalytic exploration to the latest developments in the field of neuroimaging, these fields have followed separate research trajectories at intervals, sharply diverging at times, in hopes of shedding light upon interpretations of distress, change, and recovery. While neuroscience has shed light upon the basis in neurons for such phenomena as states of emotion, memory, and mental imagery [1,2,3], psychotherapy has developed an experiential and relational vocabulary to describe the subjective aspects for these very same processes, thus highlighting the intertwined nature of meaning, narrative, and feeling within the context of human relationships [4,5]. Despite significant advances in each field, the convergence of the disciplines has been halted by an epistemological roadblock: the mind–body split typical of Cartesian dualism, establishing a disjunction between the neuronal and the psychological and between the material and the experiential [6,7,8]. When referring to narrative, we do not oppose it to science or present it as incompatible with scientific inquiry. Rather, narrative is considered here as a methodological and epistemological bridge that enables the articulation of subjective meaning-making processes within a broader scientific framework. In psychotherapy, narrative approaches are widely recognized for their ability to capture lived experience, which complements rather than contradicts empirical perspectives.

This dualistic orientation has generated theoretical schemas that overemphasize biological determinism or symbolic interpretation to the exclusion of the important realization that each model is necessarily incomplete in its rendition of the complex emergent and interactive processes that form between neural, cognitive, relational, and cultural systems. When viewed from the exclusive clinical orientation of neuroscience, there arises a disquieting possibility that psychotherapy will be viewed in isolation as merely a device for brain activation or normalization. By the same token, psychotherapeutic models that fail to take account of insights generated through neuroscientific inquiry may overlook the physical substrate through which all experience is first received and then radically redefined. The inherently systemic nature of the psyche—a mutual convergence in which synaptic activity, embodied cognition, relational engagement, and culturally embedded meaning co-regulate and co-evolve—is too often underappreciated.

In this work, we adopt the perspective that psychotherapy may be understood as a practice of meaning-making and identity reconstruction. We acknowledge that this is not a universal definition but a theoretical stance consistent with integrative and constructivist approaches. Other traditions in clinical psychology and psychiatry emphasize psychotherapy primarily as a treatment of symptoms and disorders. Our intention is not to deny this clinical orientation but to highlight the complementary epistemological potential of a systemic and meaning-centered lens.

Here, complexity theory opens up a frontier relevant to transformative epistemology. The properties inherent to complex adaptive systems, i.e., non-linearity, self-organization, and emergence, can be examined in the context of their reciprocal connections, revealing an extraordinary similarity to the healing process. The latter usually unfolds by way of non-linearity and often emerges from unexpected changes, unexpected realizations, or the slow unfoldment of behavioral and meaningful patterns [9,10,11,12,13]. Within the prevailing paradigm, psychotherapy could not be seen as a linear succession of techniques; it is instead correctly described as the co-creation of a dynamic field facilitated by the working of feedback loops, in which the therapist and the patient are equally engaged in the formation of an adaptive regulatory network. This understanding finds additional support from the recognition that successful and sustained therapeutic transformation qualifies as a reorganization of psychological structures in concordance with neural networks [3].

The understanding of complexity is advanced by cognitive science through reconceptualizing cognition as embodied, enactive, and extended. Cognition is no longer viewed through the lens of being restricted to the limitations heretofore ascribed exclusively to the brain but is seen to emerge from a multifaceted interrelation between the body, its environment, and cultural artifacts [14,15]. Psychotherapy is thus enabled to change not just internal stories but also the embodied practices and enacted social interactions that define human relationships. In this sense, psychotherapy is intimately coupled with the ecological contexts in which human lives are lived. Such intercoupling is further confirmed by findings of neuroscientific research suggesting that neural circuits are reorganized through embodied and social experience—an interrelatedness that makes it impossible to disconnect subjective mental stories from their biological underpinnings [2,4]. Our references to cognitive science follow the established interdisciplinary field that integrates psychology, neuroscience, artificial intelligence, linguistics, and philosophy, rather than implying that ‘cognition itself’ is science. We acknowledge that the boundaries of scientific status in psychotherapy remain debated, and our intention is to frame cognition as an interdisciplinary object of study rather than an unquestioned scientific certainty.

To clarify this convergence, we propose the idea of entangled autopoiesis. First described by Maturana and Varela [16], the idea applies to the inherent self-producing and self-regenerating properties found in living systems [17,18,19]. However, it is important to note that the human mind does not work in separate compartments but in connected networks involving culture, language, and technology that at the same time restrict and enable its possibilities for self-organization [20,21,22,23]. By taking an entangled autopoietic view of psychotherapy and neuroscience, we make the point that the idea of healing needs to be reconceptualized from the repair of faulty processes to the support for conditions that enable coherence, resilience, and openness to novelty in self-organizing systems—neural, psychological, or relational. Therapy thus moves from intervention in a passive brain or psyche to co-regulation within an autopoietic field.

This understanding reflects current thought in cognitive neuroscience, where the suggestion that the brain acts as an integrated system has been emphasized by the active interaction of neuronal populations in concert with physiological processes and external influences [24,25]. Also, this understanding conforms to the tenets of systems science and the field of cybernetics in postulating that autopoietic systems are by nature not isolated; their evolution and continuation depend on interaction and connectedness in larger networks of meaning [21]. Thus, psychotherapy engenders a situation ripe for the creation of autopoietic processes susceptible to influence and change by intentional relational interaction.

The field of artificial intelligence presents new possibilities alongside constituting a profound challenge. Used both as a metaphor and a tool, AI technologies enable the building of models that incorporate feedback loops and emergent patterns that reach beyond human awareness; at the same time, these technologies raise questions about the place of non-human actors in the therapeutic field. Used as diagnostic tools, pattern recognition algorithms, and simulations of relational dynamics, AI technologies have entered the therapeutic field, thus increasing the complexity of this field.

The main aspiration here is to bring psychotherapy and neuroscience together within a multidimensional framework incorporating autopoiesis, thus overcoming the constraints entailed by linear and reductionist perspectives. Throughout this paper, the term psychotherapy is used in reference to evidence-based therapeutic practices recognized in clinical psychology and psychiatry, rather than to non-validated or pseudo-therapeutic interventions. We are aware of the diversity and at times contradictory nature of psychotherapeutic schools—ranging from psychoanalytic to cognitive–behavioral and integrative approaches—and our framework does not aim to resolve these theoretical divergences. Rather, entangled autopoiesis is proposed as a metatheoretical paradigm that can provide a systemic lens for conceptualizing psychotherapy as a professionalized, evidence-grounded practice.

By way of an explanation of the enfolded theory of autopoiesis, we propose a radical recontextualization: framing psychotherapy as a continuous process entailing self-reference systems interconnected mutually, from which meanings and resilience come forth as byproducts of the interactive dynamics between neural, cognitive, relational, cultural, and technological aspects. The goals of psychotherapy would then move away from solely overcoming discrete problems in an objective world towards allowing the processes inherent to the mind in self-organization, regeneration, and transformation.

## 2. Theoretical Background and Epistemological Foundations

### 2.1. Psychotherapy and Clinical Neuroscience

The integration of psychotherapy and neuroscience has been marked by growing recognition that profound change exceeds symbolic understanding or behavioral conditioning; instead, it represents a neurobiological reorganization of adaptive human functioning. Advances based on affective neuroscience and neuropsychotherapy have made clear the processes by which constructs like neuroplasticity, the neurobiology of attachment, and stress regulation create the biological substrates for the therapeutic efficacy of psychotherapy [3,4,26]. This development highlights the intimate relationship between psychological and biological factors: psychotherapy does not function in parallel but through the brain, with effects both impacting and being impacted by neural reorganization.

A striking example of such interaction is illustrated in the case of trauma. Chronic stress and disturbances in early attachment give rise to measurable epigenetic changes and neuroplastic changes that affect the regulation of the hypothalamic–pituitary–adrenal (HPA) axis, thus heightening vulnerability to psychopathological outcomes [27,28,29]. Significantly, dysregulation of the right hemisphere has been identified as a critical mechanism underlying the development of traumatic attachment and the onset of posttraumatic stress disorder, and hemispheric specialization is a vital aspect of the regulation of affect [30]. Such evidence highlights that trauma is more than just psychological damage; it is a neurobiological marking of relational breakdown, in which the brain itself is an index of the failure of early settings to provide safety and co-regulation [31]. Such understanding makes the practice of psychotherapy inextricably bound up with the discipline of neuroscience: the mechanisms of healing and organization in therapy conform to, indeed depend upon, the capacity of neural networks to change when states of safety and trust are in place [28].

The alliance, once thought to be the main determinant of successful psychotherapy, has increasingly been described as a neuro-relational regulator [32,33]. Through attunement, co-regulation, and affect mirroring, among other mechanisms, the therapist provides external regulation to the patient’s nervous system, which dampens the stress response, lowers amygdalar activity, and activates prefrontal circuits of executive functions and emotional regulation [26,34]. Thus, therapeutic involvement goes beyond mere verbal communication or cognitive insight; it becomes a biological context in which the patient’s stress axis, memory networks, and emotional circuits are re-wired within the boundaries of the therapeutic relationship. The psychotherapist has long supposed that healing takes place within the context of relational processes, and emergent findings in neuroscience have been proven to support this contention profoundly, revealing that experiences rooted in trust, security, and significance evoke molecular and neural processes leading to increased resilience [28,33].

In parallel, it would be important to consider the limitations inherent in a solely clinical framework for neuroscience when attempting to make sense of the broader range of psychotherapeutic change. While examining the neural basis for change is important, the reduction in interrelated processes to their neurobiological components can freeze the emerging dynamics that are inherent to lived experience [35]. Emotion, memory, and self are impossible to adequately explain without regard to their existence in the form of neural activity; instead, they are located within narratives, cultures, and existential contexts that cannot be reduced [7]. The brain does not alone think, feel, or heal; rather, it exists within interactive systems and networks of meaning. Clinical neuroscience, if removed from larger contexts, risks making psychotherapy a mechanistic model, thus erecting a new type of reductionism under the guise of scientific validity.

As such, the goal is not to subjugate psychotherapy to neuroscience but to recognize their interdependence. While neuroscience explains the mechanisms by which psychotherapy causes changes in the structure of the brain, psychotherapy provides the meaning and interpersonal relationships that are essential for these changes to have therapeutic effects. As Grosjean [36] illustrates, development “from synapse to psychotherapy” must not be conceived as a straight line but as a two-way interaction, in which synaptic adaptation is both the precondition and the result of psychotherapeutic change. As such, psychotherapy should be conceived not as solely being a top-down or bottom-up intervention but as a process with interchangeably influential dynamics that simultaneously act at neural, psychological, and relational levels.

Here, the convergence of clinical neuroscience and psychotherapy requires an epistemological model that emphasizes entanglement over correspondence. This view provides a theoretical construct for therapy as a systematic neuro-relational regulation, where neuroplasticity, narrative, attachment, meaning, stress physiology, and culture are interdependent components within an integrated framework. This perspective transcends the mere augmentation of clinical technique; it also allows for an in-depth exploration of complexity science, cognitive theories, and autopoietic theory, all of which illuminate the dynamic aspects of the therapeutic process beyond reductionist constraints.

We further clarify that while supportive communication, empathy, and attunement can occur in many human interactions—between friends, family members, or professionals—psychotherapy refers specifically to structured, professional interventions delivered by trained and accredited specialists in psychology or psychiatry. We do not include informal support or pseudo-therapies within the scope of our framework.

### 2.2. Cognitive Science Perspectives

Although the biological basis for treatment lies in neuroscience, cognitive science explains the epistemological relationship between objective processes and subjective experience and thus highlights the importance of embodied, extended, and enactive cognition. The shift from classical cognitivism to enactivism has revolutionized the understanding of the human mind; it is no longer viewed as a disembodied computational machine processing input and output but as an individual, living, self-organizing system marked by bodily experiences, environmental interactions, and cultural contexts [37,38]. In this framework, cognition is distributed in the brain, body, and environment, and emerges through continuous interactions that are at once neural, affective, and social [39,40].

This diversion directly parallels the work of psychotherapy, which cannot be reduced to mere action on disembodied representations but must instead be understood as the process of redefining embodied schemas of perception, affect, and action. Cognitive schemas are interpretative frameworks in terms of which people organize experience and hence shape expectations, emotional responses, and interpersonal relationships [41]. As these schemas consolidate, become distorted, or evolve maladaptive features, they can create distress and consolidate dysfunction. Therefore, psychotherapy is seen as a schematic and narrative reorganization process that loosens up maladaptive structures and enhances adaptable and flexible models of self and the world.

Most recent narrative studies on identity reinforce understanding of the phenomenon. The self cannot be seen as the end point of an immutable construct; instead, it is constituted as a continuously revised and contextualized narrative within life stories, self-defining memories, and personal scripts. Such narratives are constructed to produce continuity and closure [42,43,44]. They shape people’s understanding of their past and their hopes for the future. Therapeutic discourse accordingly moves beyond the correction of maladaptive cognition to the practice of the re-authoring of narratives, in which experiences of trauma, shame, or failure are rewritten in narratives of survival, agency, and relational belonging [45,46]. Thus, psychotherapy becomes described as an active involvement in the co-constructive creation of identity, intertwining personal narratives within larger cultural, familial, and relational frames.

The enactive and embodied shift in cognitive science holds that the processes cannot be exclusively translated in terms of linguistic or symbolic exchanges. Meaning is constituted not merely in words but in gestures, silence, posture, affective attunement, and the tacit dynamics of the therapeutic environment per se [47]. Full-body engagement, as the result for instance in experiential or somatic modes of psychotherapy, captures the idea that cognition occurs within and through the body, so that therapeutic change emerges as much from sensory-motor recalibration as from insight ensuing from reflective thought [38,48]. The viewpoint finds support in the extended mind thesis, which clarifies that instruments, environment, and symbolic systems variably ranging from journals to media serve as constitutive parts of the cognitive process per se, acting as external scaffolding allowing therapeutic change to emerge [37,48].

A second implication of these approaches is that psychotherapy at its core is an affective and social process. Cognition cannot be seen simply as “cold” information processing but is itself affective, relational, and contextually grounded [40]. The cognitive–affective state is co-constructed by the patient and therapist, in which meaning emerges through embodied interaction and co-regulation. The inclusion of cognition, affect, and embodiment in this framework holds up the importance of working with implicit modes of communication—attunement, resonance, and embodied empathy—as fundamental processes of therapeutic transformation [38,39].

In this way, cognitive science does not simply supplement neuroscience; instead, it serves as a conceptual bridge. It makes understandable the activity in the neurons in relation to the organization of lived, bodily life, thus safeguarding the unity of the brain and the psyche. By combining embodied, enactive, and extended accounts with schema theories and research on narrative identity, cognitive science provides the needed theorizing to make psychotherapy intelligible not only as an abstract “talk therapy” but as an embodied practice in meaning-making and reconstruction of identity. It integrates the biological and the existential so that the neuroscientific description of therapeutic change remains grounded in the phenomenological quality of human existence.

### 2.3. Systems Engineering and Complexity Theory

Neuroscience and cognitive science clarify both the experiential and biological aspects of the process, and systems engineering and complexity studies provide a vocabulary that describes its organization and behavior. These offer a lexicon that includes feedback loops, adaptation, resilience, and emergent behavior—terms that are germane to psychotherapeutic process, engineered systems, and ecosystems [49,50,51]. Systems engineering, for instance, emphasizes the organization of complex processes: a recursive adjustment of input, interaction, and output that seeks to optimize performance. Applied to psychotherapy, this conceptual framework describes the psychotherapeutic process as a dynamic regulatory mechanism through which intervention creates a perturbation that induces adaptive responses at the neural, cognitive, and relational levels [52].

The dyad can be thought of as a complex adaptive system (CAS)—a network in which local interactions give rise to emergent patterns that are unpredictable when one examines the properties of individual agents [53,54,55]. Unlike linear models, which presuppose linearly determined causality, CAS models highlight the non-linear processes by which psychotherapy emerges as a consequence of recursive feedback and dynamic constraints [56]. The small-scale intervention that involves brief empathic resonance can effect large-scale reorganizations in self-concept and relational abilities, whereas large-scale interventions can dissipate without producing any observable effects. This argument documents the sensitivity to initial conditions that marks the field of complexity science, where even small differences in initial states can give rise to distinctly different outcomes [56]. As a consequence, psychotherapy, like complex systems, contains the paradox of unpredictability and patterned cohesion; namely, while the ultimate outcomes are unpredictably determined, they tend to converge towards states like resilience, cohesion, or, alternatively, maladaptive inflexibility.

Stress research provides a relevant example to guide this line of thinking. Early relationship experiences set the foundation across the life course that shapes the course of both vulnerability and resilience. Dysregulation of attachment and the stress response system, as described by Radley and others [27], forms the foundation for maladaptive behavior; yet resilience is not realized in the absence of stress but through the ability to reorganize under adversity [26,28]. It is this understanding that is consistent with Holland’s [54] conceptualization of the Complex Adaptive Systems (CAS) as systems whose evolution occurs through perturbation: here, disruption is not pathological per se but rather requires reorganization. It is within this context that psychotherapy imposes perturbation within a safe environment, thus allowing for the deconstruction of maladaptive attractant states in favor of new, adaptive patterns of regulation.

Systems engineering holds that successful regulation does not depend on hard control but on adaptive design—namely, on systems able to learn, to reorganize, and evolve in the face of new contexts [50,51]. For the therapeutic framework, this would mean the therapist acts as co-regulator and not as some external “engineer of the psyche” inasmuch as he becomes involved in system-wide adaptation. The therapist can be compared to the systems architect; he has no duty to introduce preprogrammed solutions but to create conditions for the spontaneous generation of new patterns of order. As engineers who are to build in resilience have to take emergent behavior and cascading effects into consideration, so too has the therapist to take uncertainty and emergence into consideration, approaching patterns of interaction rather than concentrating merely on individual symptomatology.

The theoretical basis for the self-organizing network thus supports the whole healing process. Resilience cannot just be seen as an outcome of treatment; it co-evolves as a vital part of the relational dynamics in which the therapist and patient affect each other through adaptive cycles. This view fits perfectly with Lansing’s description of a complex adaptive system (CAS), which involves systems that continuously create higher-order patterns at local interaction levels. In therapy, episodes of shared meaning, emotion synchrony, and narrative restructuring are all such emergent processes—these are new attractors in the patient’s psychological space.

Through the placement of psychotherapy within the paradigms of complexity theory, one moves beyond a mechanistic model of intervention and into an ecological model of co-regulation. Within this model, the therapeutic relationship itself is framed as an adaptive system that adapts to perturbations, emergence, and resilience. This point places psychotherapy in alignment with the principles of complexity science while situating it within a wider epistemological framework of living, adaptive systems. Such an epistemology provides a conceptual foundation for autopoiesis and entangled systemic integration.

### 2.4. Autopoiesis and the Living Mind

While complexity theory specifies the grammar of dynamical interaction, autopoiesis explains its ontological basis. First formulated by Maturana and Varela [16], autopoiesis refers to the ability of living systems to recursively create and renew their own organization, without external support, through renewal processes. Unlike machines that work according to externally formulated blueprints, autopoietic systems continuously reproduce the very parts that define their existence, hence securing their independence and durability in the presence of environmental disturbances. This deep insight radically reshaped the understanding of life as an autonomous organization process, shifting attention from structural fixity to circular causality and ongoing self-generation.

The idea has been radically redrawn, reassessed, and refashioned over the course of the last four decades [17,18,19]. An academic debate has grown up around the question of whether cognition belongs to autopoiesis, or whether the principles of autopoiesis are limited to biological systems, or if such principles can be extrapolated to psychological, social, and technological systems [20,21,22,23]. The current viewpoint in mainstream systems thought is that autopoiesis is not solely biological; rather, it provides a basis for understanding identity, meaning, and culture as processes that are self-organizing and self-producing. Psychological selfhood in this context can be framed as an autopoietic system that recursively builds and replenishes its thought, feeling, and narrative modes. These dynamics, already elaborated in systems engineering, are here transposed into the cognitive domain, where meaning-making emerges through recursive self-organization of perception, memory, and narrative.

From a therapeutic point of view, autopoiesis harbors the essence of psychic resilience. The patient cannot be seen as the recipient of passively given insight; instead, they are an active self-organizing system that exploits embodied, relational, and cultural affordances to produce new forms of adaptation and identity [57,58]. Here, psychotherapy functions as ecological support for autopoietic renewal: it supplies a relational structure in which maladaptive cycles—such as rigid schemata, traumatic memories, and dysregulated feeling—may be interrupted, and adaptive cycles of meaning, regulation, and narrative coherence can be fortified. This formulation fits with the findings of neuroscience, which show how therapeutic intervention elicits plastic reorganizations of circuitry in the brain [3,4], and with cognitive science perspectives that highlight how narrative and embodied experience give rise to the reorganization of selfhood [38,43].

Autopoiesis also underscores the intertwining between the individual mind and its technological and cultural environment. Koch [59] has revealed that social and spatial systems are autopoietic in character, showing that human subjects are continuously entangled in larger recursive networks of meaning. Pessoa’s [24] exploration of the “entangled brain” takes a similar position, showing that cognition and affect can no longer be severed within separate neural modules; instead, they are the result of distributed and contextually dependent networks. Such entanglement extends to the technical domain: under digital conditions, the psyche becomes increasingly co-constituted by meanings and relations to artificial intellect, virtual reality, and algorithmic networks. The mind, when framed as autopoietic, cannot be reduced to the physical boundaries of the skin or skull but must be recognized as culturally, relationally, and technologically mediated.

Within this framing, artificial intelligence (AI) does not suppress human agency but joins the autopoietic web of cognition as an external support system and reflecting mirror. Therapeutic practice informs the addition of mechanisms such as biofeedback, narrative analysis, or recognition of affects—becoming part of the recursive loops by which subjects respecify themselves. The idea of autopoiesis thus compels us to view selfhood not as an isolated essence but as a node in a web of recursive human–machine–cultural interaction [23].

The living mind, as an autopoietic system, transforms the practice of psychotherapy. Therapy is no longer seen as the imposition of order by a distant expert or merely the elimination of pathology. Rather, it is the establishment of conditions that allow the psyche to self-organize, adapt, and regenerate. This requires recursive loops that include embodied experience, relational attunement, cultural meaning, and, in today’s world, technological mediation. Autopoiesis is therefore not only a theoretical construct but also a clinical ethos: to enhance the individual’s capacity to endure as a living system, showing resilience in the ongoing self-generation of meaning and identity in the face of complexity.

## 3. The Entangled Autopoiesis Framework

The integration of psychotherapy, neuroscience, cognitive science, and systems theory leads to the theory of entangled autopoiesis—a framework that explains therapeutic change through dynamic reciprocal interactions among recursive processes at multiple levels: neurons, cognition, relationships, culture, and technology. Instead of viewing these levels in distinct ways, entangled autopoiesis describes the psyche as an autopoietic system that is deeply embedded within and co-regulated by multiple levels of interaction that recursively affect and reinforce each other [5,16,60].

### 3.1. Psychotherapy as Systems Integration

Modern integrative models of psychotherapy posit that no one particular theoretical orientation—psychodynamic, cognitive–behavioral, or interpersonal—can sufficiently capture the multifaceted nature of mental suffering and personal change. In this light, an interpersonal neurobiology approach hypothesizes that mental health is best understood as the intersection of the brain, mind, and interpersonal relationships, each of which contains important aspects of human functioning. The autopoiesis approach builds on this model by placing these domains in systemic and cultural contexts, thus implying that identity, resilience, and meaning arise as secondary effects of the interplay among neural plasticity, embodied cognition, relational attunement, and cultural–symbolic structures. According to this approach, psychotherapy moves beyond symptom relief or insight production; it becomes a process of systemic integration that involves biological, psychological, and cultural levels of organization.

### 3.2. Dynamic Reciprocity in the Therapeutic Relationship

At the center of this model of integration is dynamic reciprocity. The patient and the therapist are complementary positions within a self-organizing relational system, rather than being clearly distinguished, such that transformation emerges from recursive cycles of interaction [61]. Empirical work has shown that synchronization of affect, physiology, and behavior—demonstrated, for instance, through speech rhythms, bodily motion, or brain function—increases the therapeutic alliance and can predict positive outcomes [62,63]. This viewpoint is consistent with Hughes’ [64] description of the rhythmicity of interaction in child psychotherapy in which reciprocity is a co-constructed flow, not a one-way process.

Philosophically, the ethic of reciprocity answers the problem of asymmetry and mutuality in therapeutic relations [65]. While the therapist and client are in different roles, change relies on a reciprocal regulation that breaks past hierarchical limitations. The supervisory situation illustrates the same process in parallel dynamics in that supervisory relations reflect therapeutic interactions and illustrate the fractal and recursive nature of therapeutic systems [66]. Autopoietically, the dyad engaged in a therapeutic relationship forms an autonomous network of relations, in which reciprocal adjustment generates emergent properties—i.e., safety, empathy, and trust—that neither participant can generate independently [67].

### 3.3. Emergence of Meaning and Resilience

At the center of entangled autopoiesis is the creation of meaning and resilience. Meaning creation lies beyond mere cognition; it is a systemic product that arises from the interplay between neuronal processes, embodied schemata, relational attunement, and cultural stories [68]. As described by Ryff [69], resilience is more than a return from adversity; it includes the promotion of self-realization and eudaimonic meaning. Case study research demonstrates that the meaning-making process is inherently multi-perspectival, allowing people to reinterpret adversity as a source of growth and reorganization [68,70].

Thus, resilience is not a construction of external intervenors but emerges as an autopoietic reorganization of the self in relation to relational and cultural contexts [71,72]. Psychotherapy, as entangled autopoiesis, is the incubatory context for the development of resilience: it deconstructs pathologic states of attractors, provides scaffolding for the reconstructions of narratives, and facilitates adaptive meanings and belonging loops [69,72]. Accordingly, on this view, resilience and meaning should not be thought of as discrete “outcomes,” but as emergent properties of a single, relationally entangled system.

### 3.4. Artificial Intelligence as Feedback and Pattern Detection

The introduction of artificial intelligence (AI) adds a new dimension to the complex autopoietic system. AI devices—ranging from neuroimaging pattern recognition to real-time emotion detection—act as feedback mechanisms that augment therapeutic insight. Developments in neuroimaging, when married to deep learning, allow for modeling of affective states and dysregulation patterns with a degree of comprehensiveness never before seen [73]. In addition, AI systems trained in multimodal data such as EEG, HRV, and GSR can provide accurate feedback for neurofeedback therapy and thereby assist in adaptive regulation [74].

In addition to quantification, artificial intelligence contributes powerfully to predictive modeling of treatment trajectories. Machine learning algorithms have been shown to forecast the mechanisms by which children regulate their mental health, thus enabling tailored interventions [75]. Shi [76] highlights the contribution of AI adaptive learning systems to emotional regulation, arguing that adaptive therapeutic interventions can dynamically modify to respond to patients’ shifting needs. Similarly, Omiyefa [77] argues that the goal of AI in precision psychiatry is to establish predictive models of treatment aiming to predict dysregulation before clinical deterioration occurs.

Such uses require a reflexive response; the therapist needs to consider artificial intelligence as a complement rather than a replacement for autopoietic control, acting as a mirror revealing hidden patterns in the feedback and allowing the system to evolve. As explained by Lorenz, Weiss, and Hirche [78] in their discussion on social robotics, reciprocity and synchrony are fundamental prerequisites in the achievement of therapeutic trust—factors with which artificial intelligence needs to replicate in order to work well as partners in clinical intervention. Used appropriately, artificial intelligence becomes a technological node in the framework of therapy, acting through recursive processes of evolution and feedback while allowing human agency to be maintained.

The Entangled Autopoiesis Framework suggests that psychotherapy is an integrative system of several levels where the psyche is an autopoietic core sustained by recursive loops between neural, cognitive, relational, cultural, and technological aspects. This framework is based on the organizing principles of complexity science and systems theory as applied to the practice of psychotherapy, underscoring the fact that change arises not from linear causation but from dynamic reciprocity and emergent self-organization [8,16,79,80].

On the biological level, psychotherapy works through the modulation of neuroplastic processes, defined as the brain’s ability to reorganize as a function of new experience. The research suggests that the neuroregulation of stress, memory reconsolidation, and attachment neurobiology are the basis for therapeutic change [4,26,27]. Dysregulation of the stress axis, especially after early trauma, sets the ground for vulnerabilities in interpersonal and affect regulatory functioning [29,30]. Within the context of the therapeutic relationship, relational safety promotes stress physiology downregulation, thus enabling reorganization of neural circuits. Note that these circuits are not isolated; their plasticity is moderated by cognitive meaning-making and relational attunement, thus making them parts within a larger autopoietic system [5,60].

Cognitive science contributes by highlighting the embodied, enactive, and extended nature of cognition [37,39,47]. Mental schemas shape how individuals perceive and respond to the world, but when rigid or maladaptive, they reinforce cycles of distress [41]. Psychotherapy facilitates schema reorganization and narrative re-authoring, enabling patients to reconstruct their life stories and integrate traumatic memories into coherent identities [43,44,45]. Narrative identity research suggests that resilience is not simply biological recovery but also a narrative process of self-renewal, grounded in autobiographical meaning-making [46]. In the entangled autopoiesis framework, cognition mediates between neural processes and cultural narratives, bridging biology with subjective experience.

The therapeutic relationship has been described as a self-organizing system with reciprocity, thus promoting healing via mutual regulation. Synchrony in emotion expression, posture, and even neuronal activation is a key factor in promoting empathy and participation [62,63]. Reciprocity has been identified as an essential process within therapeutic relationships that promotes cooperation and trust [61,67]. The interconnected autopoiesis model describes the therapist and client as nondifferentiated nodes that demonstrate interdependence, leading to the development of relational properties such as trust and protection [64,65,66]. This type of reciprocity fits perfectly into Siegel’s [80] interpersonal neurobiology framework, where mental health is described in terms of the interconnection between the brain, the mind, and interpersonal relationships.

Psychotherapy cannot be understood outside of the cultural–symbolic contexts in which people live. Collective narratives and symbolic resources that promote continuity and a sense of belonging have significant effects on identity and resilience [69,81]. Resilience, in this regard, appears as a byproduct of the processes of meaning-making that take place when adversity is reconstructed in relational and cultural contexts [68,71,72]. For this reason, psychotherapy is conceptualized as a meaning-making process hosted within a particular culture, placing individual suffering in larger symbolic contexts and allowing people to build self-sustaining yet socially grounded identities [70]. Through narrative incorporation in the autopoiesis processes, therapy helps build systemic resilience.

In modern contexts, artificial intelligence (AI) is an innovative player in autopoietic systems. Neuroimaging and affect-detection tools, aided by AI, provide rich feedback regarding psychophysiological state and enable adaptive control [73,74]. Machine learning algorithms are equipped to predict dysregulation and provide tailored intervention strategies, thus optimizing accuracy in mental health intervention [74,76,77]. Instead of replacing therapists, AI is another mediator, thus optimizing the capacity of the system to identify hidden patterns and adaptive dynamics. As described by Lorenz, Weiss, and Hirche [78], reciprocity and synchrony are universal constituents, even of social robots; the same is true for AI in psychotherapy, which is required to adapt to the relational nature of care. In the explanatory framework of autopoiesis, AI is not regarded as an external intervention but as a technological node in the recursive system, thus extending the scope of feedback, monitoring, and adaptation.

There arise from these interlinked loops meaning, resilience, and the redefinition of identity—attained not by the action of an external force but through the autopoietic character of the system itself. Thus, psychotherapy becomes a central hub of systemic integration: a center where biological, cognitive, relational, cultural, and technological aspects intersect in order to enable the reparative mending of the human psyche.

## 4. Clinical and Practical Implications

An embracing of psychotherapy via the lens of entangled autopoiesis requires a deep shift in therapeutic practice. Therapy can no longer be conceived merely as the application of preordained techniques but rather as the tuning of feedback loops that engage cognition, emotion, and neurophysiology. Change is recursively mediated along biological, psychological, relational, and cultural planes. Within this vision, the therapeutic relationship is viewed as an adaptive network that dynamically develops over time, based on the processes of synchrony, reciprocity, and mutual regulation [61,62,79,80]. Therapeutic dialog creates emergent properties—such as trust, resilience, and coherence—that cannot be assigned to any individual participant; these properties instead arise from their reciprocally organized system of interaction [64,65].

Such a systemic orientation has important clinical applications. As concerns trauma treatment, intervention can be framed as attempts to disrupt maladaptive attractor states and break up rigidly organized neurocognitive circuits that are based on dysregulated systems of stress, while simultaneously establishing new adaptive cycles for security and regulation [26,29,30]. For anxiety and depressive disorder, treatment intervention entails the re-patterning recursive interaction between neural circuits involving withdrawal or fears and the narrative structures that sustain maladaptatative identities [3]. Psychotherapy, in this conceptualization, is framed as a process of reconstruction of the self, in which new narratives and schemata are interfaced with neural and relational change to enable recovery in autopoietic wholeness [43,81]. The therapy is no longer framed in linear or symptomatically directed terms; instead, it is reconceptualized as the facilitation of contexts for self-organization, adaptive transformation, and reconstruction of the psyche at multiple systemic levels.

The integration of artificial intelligence as a co-regulator sets a systematic framework for future clinical domains. By simulating psychophysiological mechanisms, like electroencephalogram (EEG) patterns, heart rate variability (HRV), or galvanic skin response (GSR), AI-enabled systems can provide instant feedback that reinforces the comprehension of the individual and fortifies the decision-making process of the therapist [74]. Improvements in machine learning allow for the detection of subtle patterns associated with dysregulation and the prediction of the following emotional state, thus providing the basis for adaptive digital systems to adjust interventions in a responsive manner [73,76,77]. For example, predictive models have been used to predict adolescent coping strategies used in emotionally relevant contexts, thus providing the foundation for personalized intervention corresponding to certain behavioral patterns [75]. In the therapeutic setting, such devices have the potential to provide complementary information for the therapist, acting like a technological mirror that discloses hidden dynamics of feedback in the therapeutic relationship.

The incorporation of artificial intelligence into the field of psychotherapy is not free of inherent dangers. Ethical considerations of autonomy, trust, and depersonalization require critical examination. While artificial intelligence has the ability to recognize and replicate patterns, it does not have the capability to replace the human qualities of empathy, presence, and attunement that form the basis of therapeutic reciprocity [78]. A reliance on algorithmic feedback threatens to reduce complex human experience to quantifiable terms, and thus unwittingly advance reductionism. The dilemma lies in using artificial intelligence as an adjunct to the autopoietic system, augmenting but never substituting the therapist–patient relationship. When reflexivity is used, artificial intelligence can augment the therapeutic process by amplifying the system’s potential for feedback, monitoring, and adaptive reorganization while maintaining the primacy of human connection.

These conceptual changes have profound implications for clinician education. Since psychotherapy is defined as the systemic construction of meaning, resilience, and regulation, it is crucial that psychotherapists are trained in the fundamentals of systems thinking, complexity science, and neuroscience [8,60]. The future psychotherapist can, therefore, be seen as the systems engineer of the psyche, capable of effective operation of recursive feedback systems, the recognition of emerging dynamics, and the creation of adaptive reconstructions. The curriculum, therefore, that will be developed for the next generation of psychotherapists will have to encapsulate the significance of interpersonal neurobiology, complexity theories, and cultural psychology, thus interdisciplinary literacy so that therapists can move unproblematically across different domains [81]. It does not disempower clinical expertise but expands it so that practitioners are able to address the full complexity of the autopoietic human psyche in biological, cultural, and technologically saturated contexts.

To illustrate how the entangled autopoiesis framework may inform practice, consider two brief examples. In trauma therapy, healing can be approached not only as neural regulation but also as narrative reintegration and relational reconnection, where therapists and clients co-create new systemic coherence. In contrast, a case of professional burnout highlights how digital overexposure, embodied stress responses, and organizational culture intertwine; here, interventions guided by entangled autopoiesis address both the individual’s regulation strategies and the systemic conditions that sustain imbalance.

Overall, the clinical relevance of the entangled autopoiesis framework highlights psychotherapy as a site for systemic convergence, the co-regulator and facilitator of feedback in artificial intelligence, and the importance of clinical training as interdisciplinary systems literacy. Together, these developments foresee a future in which the therapy will no longer be viewed as a one-way linear intervention; instead, it will be framed as an adaptive, autopoietic, multidirectional process responding to the recursive dynamics between the brain, mind, relationship, culture, and technology.

## 5. Epistemological and Methodological Challenges

The hypothesis of psychotherapy as an enmeshed autopoiesis not only introduces the promise of new therapeutic possibilities but also creates significant epistemological and methodological problems. Most significant of these problems is the threat of reductionism, which appears when metaphors developed in systems engineering are transposed to the therapeutic relationship. Although metaphors like feedback, regulation, and emergent behavior are useful tools for understanding therapy as a systems phenomenon, there is the hazard that these metaphors can reduce living experience to a mechanistic model. Patients should not be conceptually grasped as machines to be tuned, and the dynamics of therapeutic engagement cannot be diminished when analogies developed in engineering are used. As Toomey and Ecker [7] warn, a misuse can occur in neuroscience and allied disciplines if subjectivity, narrative, and cultural meaning are overshadowed by an overemphasis on biological correlates. Similarly, uncritical and unconditional borrowing of systems metaphors risks hiding the complex, multifaceted representations of the embodied, complex experiences of human suffering and transformation.

To solve this problem, we need to strive for interdisciplinary integration as opposed to depending on separate fields of expertise. There should be ongoing communication between psychotherapy, neuroscience, cognitive science, and systems theory so that each contributes its particular take while also acknowledging the limitations of the others. According to Constant, Badcock, Friston, and Kirmayer [8], the future of psychotherapy and psychiatry relies on a multilevel systemic comprehension that integrates evolutionary, cultural, and computational aspects without favoring one dimension over the others. This synthesis requires that clinicians and researchers develop interdisciplinary literacy—a proficiency that allows them to move fluidly between biological, psychological, relational, and cultural levels of analysis without confusing these distinct domains.

Methodological advances are necessary to realize this vision. Computational psychiatry is a promising direction, using Bayesian and predictive coding formalisms to make inferences about how neural processes map onto cognitive and behavioral dynamics. These methods allow researchers to think about psychiatric symptoms as inferential problems within adaptive systems, thus revealing possibilities for personalized diagnostic testing and treatments according to autopoietic principles. Network analysis of symptom dynamics is also another novel strategy; instead of defining psychiatric disorders as fixed entities, this method envisions symptoms as nodes in interconnected networks that have dynamic properties, enabling clinicians to track changes in psychological organization over time and across contexts [13]. The same interconnected autopoiesis framework applies to this perspective as well, in that change can be described in terms of holistic system reorganization, as opposed to linear correction.

Another methodological challenge arises from the combination of hybrid psychophysiological and relational measures. Traditional clinical research usually balances biological measures, for example, EEG, HRV, or fMRI, or narrative-relational accounts, but the autopoietic method requires us to consider both areas equally. The most recent developments in multi-modal assessments have facilitated the combination of psychophysiological monitoring and analysis of the discourse of therapy, synchrony, and narrative change. Hybrid measures would allow researchers to relate the co-evolution of brain plasticity, regulatory processes for the affects, and narrative identity over the course of the dyadic interaction trajectory and thus gain a better understanding of the recursive interaction between meaning and biology.

Ultimately, the entangled model in autopoiesis requires investment in reflexivity, which implies recognition of the epistemic limitations inherent within the enterprise of characterizing complex living systems. Autopoiesic systems are, by definition, self-producing and self-maintaining; accordingly, external attempts at differentiating or modeling such systems will be partial and tentative. One needs to be ever-vigilant to the threats in the direction of epistemic overreach: while computational psychiatry, network analysis, and technology-augmented systems for feedback provide invaluable information, they do not encompass the lived, embodied, and relational aspects of psychotherapy. As aptly described by Northoff, the neural correlates to consciousness and emotion cannot simply be translated into neuronal patterns but rather are constituted in the fields of culture, relationship, and existence.

The relevance of methodology within the entangled autopoiesis framework has two essential aspects: first, the quest for new, integrative modes to chart systemic processes at various levels; and second, the fostering of reflexive humility in the understanding that the psyche as an autopoietic living system will always move beyond the representations we make about it. Thus, psychotherapy is described simultaneously as a science and an art; it is a recursive integrative practice that needs to avoid reduction while at the same time celebrating complexity.

**Figure 1:**
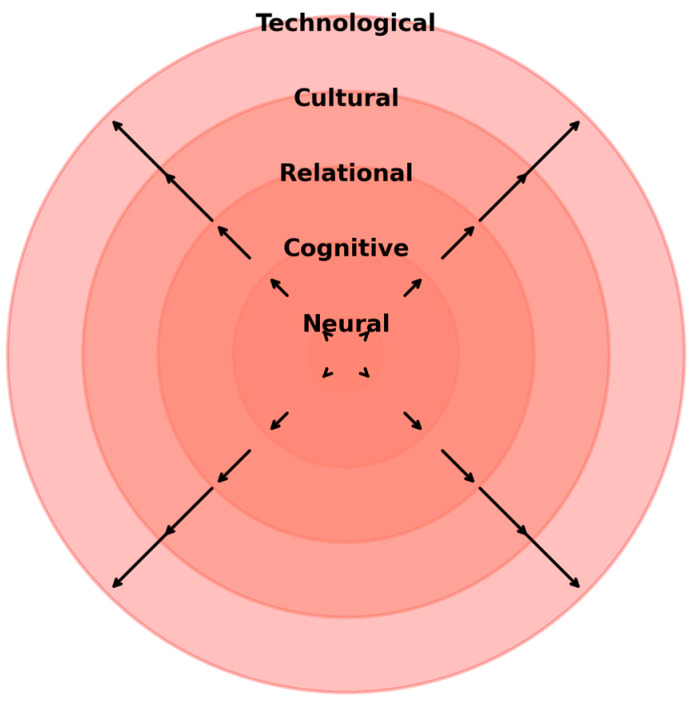
The entangled autopoiesis framework: systemic dimensions and recursive interactions.

Figure 1 provides a conceptual mapping of the entangled autopoiesis framework, situating therapeutic processes across neural, cognitive, relational, cultural, and technological dimensions, with recursive arrows indicating their entanglement.

## 6. Discussion

We acknowledge that the concept of autopoiesis has been applied in psychotherapy theory before. The novelty of our approach lies in the entangled extension of autopoiesis, which situates psychotherapy within recursive systems that are both biological and cognitive, while integrating complementary perspectives from neuroscience, artificial intelligence, and systems engineering. In this way, entangled autopoiesis does not replicate existing applications but repositions them within a broader interdisciplinary epistemology.

The entangled autopoiesis theory thus makes an important addition to the current debate between psychotherapy, neuroscience, cognitive science, and systems theory. Compared to other prevailing integrative paradigms—in particular, the biopsychosocial model, the neurocognitive approaches, and the systemic family therapy schools—this one integrates not merely the coexistence but also the recursive interrelatedness of different analytical levels. Whereas the biopsychosocial model in itself is revolutionary, it has the danger of being used as a checklist consisting of discrete domains, each working independently to enhance health without generating reciprocal interaction. Neurocognitive models emphasize the complementarities between brain and behavior; however, they do not address the synergic relations between relational and cultural mediating processes. While systemic approaches ring true in their resonance to family and relational processes, they are grounded neither in neurobiology nor in cognitive science. The entangled autopoiesis theory seeks to bring these dissonant views together by advancing an ontology for living systems that would ideally accommodate the processes at the levels of the brain, cognition, relationship, culture, and technology as mutually constitutive self-organization loops [8,79,80].

It is also important to note that the entangled autopoiesis framework resonates with the broader movement of psychotherapy integration and unification. As integration efforts seek to reconcile different schools of therapy under shared principles and practices, entangled autopoiesis provides a systemic and complexity-based lens through which such unification can be further conceptualized. By situating psychotherapy within recursive and multi-level processes, our approach aligns with the integrative aim of creating a more comprehensive, flexible, and responsive therapeutic science.

Here, psychotherapy is reconceptualized not simply as a remedial treatment to treat particular symptomology but as an interactive process allowing tuning and balancing in regard to resilience, thus allowing for meaning-making. Complexity science teaches that adaptive systems attain stability not by virtue of rigid control processes but by dint of mobility, reorganization, and feedback processes. Likewise, psychotherapy works not by eradicating pathology but by establishing conditions allowing innovative modes of coherence and resilience to emerge in response to perturbations. Trauma therapy illustrates the principle well; destabilization of maladaptive attractor states—such as hyperarousal or dissociation—allows the formation of new patterns of regulation and meaning, reinforced by recursive processes of relational attunement, neural plasticity, and narrative reconstruction. By positing resilience as an emergent property of systemic integration, the intertwining autopoiesis approach locates psychotherapy within the larger ecology of living systems.

This approach also intersects with several prevailing epistemological streams. First, it is in line with the school of the embodied mind, which denies disembodied cognition and sees the mind as embodied in the interactions of body, environment, and culture [37,47]. The entangled autopoiesis idea extends this view by placing embodiment in recursive systemic processes that include relational as well as technological mediation. Second, it corresponds with second-order cybernetics, which places the observer as part of the system being observed. In clinical situations, the therapist does not function as an external agent of change but instead as a co-regulator within the system, whose presence further shapes emergent outcomes [65,66]. Third, it draws from the school of clinical neuroscience epistemology, which, as described by Siegel, seeks to understand the mind as an integrated whole where genetics, brain maturation, attachment, and culture collectively form mental health [80]. The entangled autopoiesis approach combines these aspects into a model that not only explains but also catalyzes therapeutic change through systemic integration across interdependent systems.

In the next decade, the incorporation of artificial intelligence is both a challenge and an opportunity. Recent developments in neuroimaging, affect recognition, and predictive modeling [73,74,75,76,77] indicate the possibility of AI serving as an adaptive element within the therapeutic system, thus increasing the effectiveness of the system in feedback, monitoring, and anticipation. In the complex dynamics of the autopoiesis model, AI moves beyond being an external device, becoming a constituent of the autopoietic network that regulates therapeutic control, engaging in the recursive processes that create meaning and resilience. The future of psychotherapy can thus be imagined as a complex adaptive ecosystem where human and technological agencies work together for the modulated, co-created, and nurtured maintenance of emergent processes for healing. But this development has to be accompanied by a reflexive commitment to ensure that the relational and cultural aspects, which are constitutive of the very nature of psychotherapy, are retained.

At the same time, bringing AI into the therapeutic space calls for a careful and nuanced ethical reflection. The essence of psychotherapy lies in preserving human presence and autonomy, and digital tools should serve as companions to, not substitutes for, this relational process. There is a real danger that technology, if used uncritically, can flatten the depth of human experience or create a sense of depersonalization. The task of the therapist, therefore, is to hold responsibility for how and when AI is used, ensuring that it supports rather than overshadows the client’s story, and that every intervention remains transparent, ethically grounded, and rooted in the lived, embodied experience of the person in therapy.

The entangled autopoiesis approach is rooted in the additive integration model, yet it moves further toward an epistemology of psychotherapy that is sensitive to and shaped by complexity. It shows that healing cannot be reduced to the mechanics of brain rewiring or the crafting of new narratives alone but emerges as a new systemic coherence, woven through recursive entanglements of biological, psychological, relational, cultural, and technological processes. From this perspective, psychotherapy is not declared to stand at the forefront of science but rather invited into an ongoing dialog with contemporary scientific disciplines—neuroscience, systems theory, cognitive science—that can enrich its language and practice. Entangled autopoiesis seeks to hold this conversation open, offering a way for psychotherapy to remain conceptually alive, empirically attentive, and deeply responsive to the human condition in a world marked by accelerated cultural and technological change.

We recognize that the contemporary domain of psychotherapy is characterized by a multiplicity of schools, methods, and professional practices, some of which are contradictory or unevenly supported by evidence. Entangled autopoiesis does not aim to erase these differences but to offer a metatheoretical lens that can integrate diverse practices under a systemic, recursive understanding of human change processes.

## 7. Conclusions and Future Directions

The article has presented the idea of entangled autopoiesis as a lens for the reconceptualization of psychotherapy, neuroscience, and cognitive science and, most importantly, for the articulation of complexity and systems theory. Crucial to the argument here has been the recognition that the mind is an autopoietic and entangled construct—a living, self-creating entity that maintains itself not in separation but in recursive interaction with neural circuits, embodied cognition, relational networks, symbolic–cultural systems, and technological intermediaries. With this framework, psychotherapy has been reframed as a practice driven by complexity, going beyond the reductionist binary between mind and body yet also respecting the complicated tapestry of human existence [16,24,80].

From this viewpoint, psychotherapy ought not to be defined as a linear treatment or as a unilateral transfer of knowledge by the therapist to the client. Rather, it is better depicted as an ecological process of systemic integration, in which meaning, resilience, and identity arise out of the co-structuring of recursive feedback loops. This development requires a move away from traditional theoretical assumptions, since therapy is becoming seen as an adaptive and dynamic process of renewal, orchestrated not by prescriptive control but by the principles of facilitation of self-organization [8]. The therapeutic alliance itself is an example of a complex adaptive network, in which reciprocity, synchrony, and mutual regulation lead to the emergence of qualities such as safety, trust, and coherence.

The model also calls for innovative pedagogy in clinical training and methodological creativity. The psychotherapist of the next generation is not only expected to have mastery of psychodynamic or cognitive–behavioral interventions but also to demonstrate interdisciplinary literacy embracing interpersonal neurobiology, complexity science, cultural psychology, and technological mediation [81]. Therapeutic training must prepare practitioners to function as systems engineers of the psyche, skilled in recursive feedback structure provision and able to develop systemic resilience. At the same time, the evolution of computational technology—from network models of symptom dynamics to AI-based systems amenable to feedback—opens up new possibilities for practical application of the entangled autopoiesis model, while also raising questions regarding its bounds with respect to epistemological scope [74,77,82].

A number of future research avenues cut through as important in the near future. First, empirical inquiry is needed to study the notion of entangled autopoiesis using network models in the domain of psychotherapy, elaborating on the co-evolution of neural, cognitive, and relational processes over time. Second, artificial intelligence-based feedback systems may improve therapeutic processes, especially with regard to the detection of dysregulation and the facilitation of resilience, but these systems require careful investigation of their ethical, relational, and cultural connotations [73,76]. Third, longitudinal clinical studies are needed to track the recursive and emergent process of therapeutic change, moving beyond symptom reduction to study how identity, meaning, and resilience are reconfigured over time and across diverse contexts.

Entangled model of autopoiesis puts forward a new epistemological viewpoint for psychotherapy; it understands the human mind to be a living adaptive system that has intimate relation to biology, culture, and technology. The model requires clinicians, researchers, and theorists to move beyond reductionist tendencies and embrace a healing framework that recognizes complexity, where resilience and meaning are not derived from external intervention but rather are the result of the self-organizing processes inherent to life itself. By adopting this strategy, it opens up the possibilities for a type of psychotherapy that not only integrates scientific understanding but also deeply understands dynamics of human existence in the process of technological mediation.

## Data Availability

No new data were created or analyzed in this study. Data sharing is not applicable to this article.
